# Supplementary material for: Evidence supported by Mendelian randomization: impact on inflammatory factors in knee osteoarthritis
Source: Front Med (Lausanne). 2024 May 28;11:1382836. doi: 10.3389/fmed.2024.1382836 (PMC11165061; doi:10.3389/fmed.2024.1382836)
Supplement: Supplementary file 1 [file Data_Sheet_1.ZIP › data/GCST90274793_5e6/793.forest.pdf]

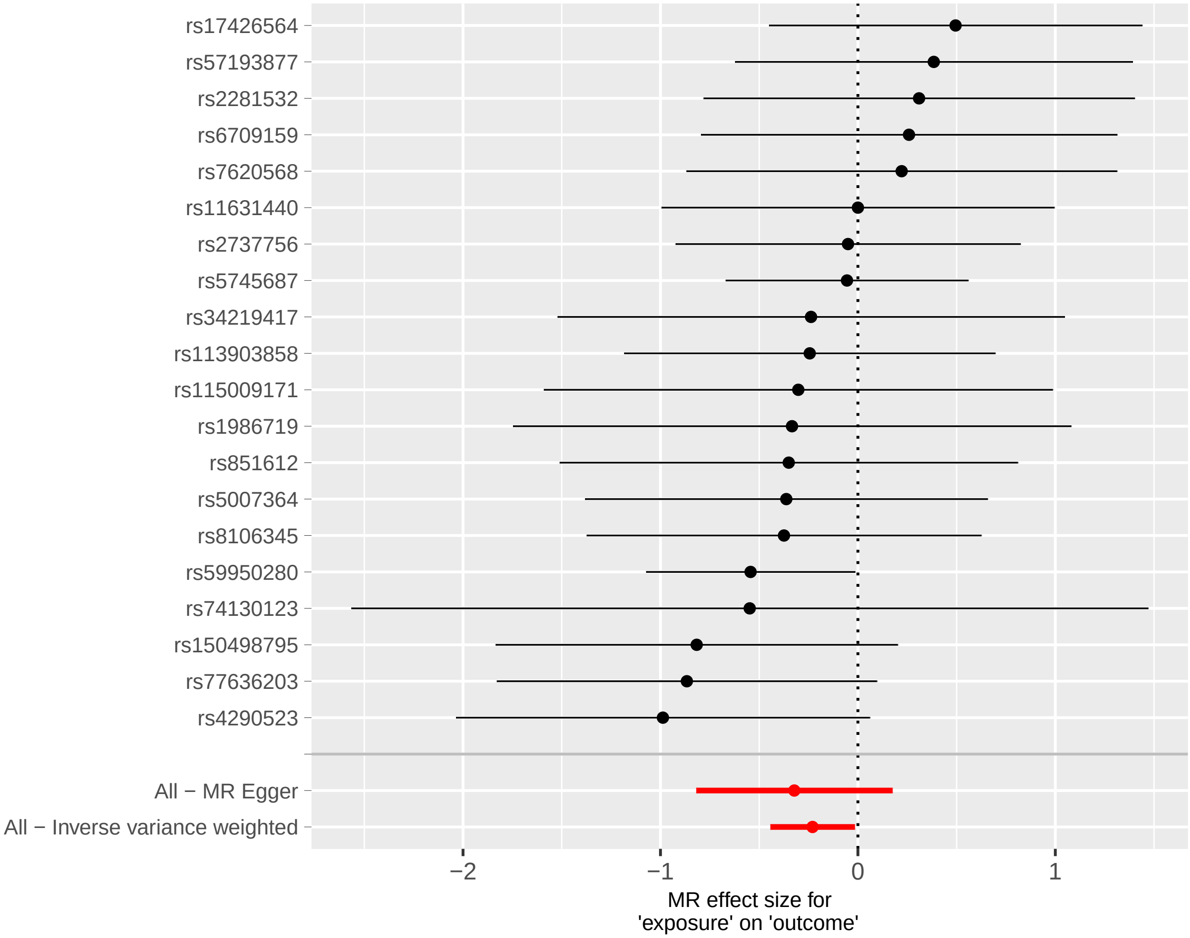
rs17426564
rs57193877
rs2281532
rs6709159
rs7620568
rs11631440
rs2737756
rs5745687
rs34219417
rs113903858
rs115009171
rs1986719
rs851612
rs5007364
rs8106345
rs59950280
rs74130123
rs150498795
rs77636203
rs4290523
All − MR Egger
All − Inverse variance weighted
−2
−1
0
1
MR effect size for
'exposure' on 'outcome'
